# DNA repair gene polymorphisms and risk of chronic atrophic gastritis: a case-control study

**DOI:** 10.1186/1471-2407-11-440

**Published:** 2011-10-11

**Authors:** Bernd Frank, Heiko Müller, Melanie Nicole Weck, Norman Klopp, Thomas Illig, Elke Raum, Hermann Brenner

**Affiliations:** 1 Division of Clinical Epidemiology and Aging Research, German Cancer Research Center, Heidelberg, Germany; 2 Institute of Epidemiology, Research Centre for Environment and Health, Neuherberg, Germany

## Abstract

**Background:**

Recent studies have reported associations of DNA repair pathway gene variants and risk of various cancers and precancerous lesions, such as chronic atrophic gastritis (CAG).

**Methods:**

A nested case-control study within the German population-based ESTHER cohort was conducted, including 533 CAG cases and 1054 controls. Polymorphisms in eleven DNA repair genes (*APEX1*, *ERCC1*, *ERCC2/XPD*, *PARP1 *and *XRCC1*), in *CD3EAP/ASE-1 *and *PPP1R13L *were analysed.

**Results:**

No association was disclosed for any of the analysed polymorphisms. Nor did stratified analyses according to ages < 65 and ≥ 65 years show any significant association with CAG risk.

**Conclusions:**

The results of this large German case-control study do not reveal associations of DNA repair pathway polymorphisms and risk of CAG. On the basis of a large number of CAG cases, they do not support associations of DNA repair pathway SNPs with CAG risk, but suggest the need of larger studies to disclose or exclude potential weak associations, or of studies with full coverage of candidate genes.

## Background

Chronic atrophic gastritis (CAG) is a well-established precursor lesion in the aetiology of intestinal gastric cancer (GC), the most common type of GC [1]. Unlike the diffuse type of gastric carcinoma, a recent steady decline in incidence has been observed for the intestinal type [1,2]. Several changes have been identified as precursors to the intestinal type of gastric carcinoma, representing sequential steps in the precancerous process: non-atrophic gastritis, CAG (gland loss), metaplasia and dysplasia [1]. This progression usually takes decades, providing excellent options for timely detection and intervention at precancerous stages [1,3]. The multistage model of gastric carcinoma development assumes that carcinogenesis is initiated by host-inflammatory response following infection by the Gram-negative bacterium *Helicobacter pylori *(*H. pylori*), and by dietary exposure to salt and nitrate, which cause DNA damage [4-7]. To date, three molecular mechanisms, by which *H. pylori *may provoke a loss of genomic integrity and promote transformation, are postulated [6,7]. These include a) mutations in mitochondrial DNA, b) the induction of a transient mutator phenotype, resulting in mutations in the nuclear genome, and c) increased amounts of reactive oxygen species (ROS) in gastric epithelial cells that induce oxidative damage in the DNA coupled to the decrease of repair activity [6,7].

The consequences of DNA damage are manifold and generally adverse. Thus, acute effects arise from a disturbed DNA metabolism, inducing cell cycle arrest or apoptosis, while long term effects from irreversible mutations may contribute to carcinogenesis [8]. Four major DNA repair pathways have been described: 1.) base excision repair (BER), 2.) nucleotide excision repair (NER), 3.) mismatch repair (MMR) and 4.) double-strand break repair (DSBR) [8]. Recent epidemiologic studies have essentially examined BER and NER pathway gene variation and risk of cancer development, disclosing associations with glioma, colorectal, prostate, lung and gastric cancers [9-18] as well as with precancerous lesions, such as colorectal adenomas or CAG [19,20].

Therefore, we sought to evaluate the relationships between putative functional single nucleotide polymorphisms (SNPs) in *APEX1 *[19], *ERCC1 *[11,12,16], *ERCC2/XPD *[10,11,13,14,17,20], *PARP1 *[9-11,19] and *XRCC1 *[15,18,19], and in *CD3EAP/ASE-1 *[12] and *PPP1R13L *genes [12], which are components of a high-risk locus between *ERCC1 *and *2 *on chromosome 19q.13.3 [12], and the risk of CAG.

## Methods

The present study comprised a subsample of the German population-based ESTHER cohort study, including 533 serologically defined CAG cases without GC history and a number of 1054 age- and sex-matched controls. Details of the ESTHER study design have been described previously [3,21]. Briefly, 9,953 women and men aged 50-75 years were recruited between July 2000 and December 2002 by their general practitioners during a general health check-up in Saarland, a federal state in the south-west of Germany [3]. The study was approved by the ethics committees of the medical faculty of the University of Heidelberg and the medical board of the state of Saarland.

According to the study protocol and informed consent, serum and blood samples were obtained from all participants. Serum concentrations of pepsinogen (PG) I and II were measured by ELISA (Biohit, Helsinki, Finland). CAG was defined by applying the most frequently used serological definition, being PG I < 70 ng/ml and PG I/PG II < 3 [3]. For sensitivity analyses, we used alternative cut-points to delineate CAG [(PG I < 70 ng/ml and PG I/PG II < 4.5) as well as (PG I < 70 ng/ml and PG I/PG II < 2)].

In line with recent epidemiologic studies that have revealed associations of BER and NER pathway gene variants with risk of glioma, colorectal, prostate, lung and gastric cancers and their precursors [9-20], selection was focused on SNPs in DNA repair genes. Non-synonymous and putative functional SNPs were of particular interest. Therefore, we searched public literature resources and databases (NCBI PubMed and dbSNP), favouring genes and polymorphisms with previous findings in view of susceptibility to precancerous lesions and different types of cancers. SNP selection included four BER gene variants (APEX1 D148E rs1130409, *PARP1 *-17G > C rs907187 and V762A rs1136410, *XRCC1 *-77T > C rs3213245), five SNPs in NER genes (ERCC1 N118N rs11615, ERCC2/XPD K751Q rs13181, D312N rs1799793, R156R rs238406 and -114C > G rs3810366), and two SNPs in CD3EAP/ASE-1 (rs735482) and PPP1R13L (rs6966) which, together with ERCC1 N118N rs11615, represent the high-risk haplotype on chromosome 19q13.3 [12].

Genotyping was performed with iPLEX^® ^single base primer extension and matrix-assisted laser desorption ionisation time-of-flight mass spectrometry (Sequenom, San Diego, USA) [21], and a random sample of > 5% was analysed twice for quality control.

Genotypes of participants were used to estimate allele frequencies, and departure from Hardy-Weinberg equilibrium (HWE) in controls (*P *≤ 0.01) was assessed using Pearson's chi-squared test. SNP associations were evaluated using unconditional logistic regression models to estimate sex- and age-adjusted odds ratios (ORs) and 95% confidence intervals (CIs). As CAG strongly increases with age [3], subgroup analyses included stratifications according to ages < 65 and ≥ 65 years.

For reasons of statistical power, we restricted our analyses to SNPs with minor allele frequencies (MAFs) > 10%, according to dbSNPs HapMapCEU data http://www.ncbi.nlm.nih.gov/snp/. MAFs of the chosen SNPs ranged from 13% - 49%. The statistical tests were implemented with SAS (SAS Institute Inc., Cary, USA), and power calculations were employed with the power and sample size software PS [22], applying the observed genotype frequencies, respectively. Two-sided Fisher's exact tests were used to compare carrier frequencies between CAG cases and controls with a type I error probability of α = 0.05.

## Results

Of 9,444 ESTHER participants with available PG concentrations (94.9%), 533 met the serological definition of CAG and were selected for this study together with 1054 controls [3,21]. Among the analysed individuals, the majority (58.2%) were females, and median ages were 65 and 66 years for women and men, respectively. Smoking was evenly distributed among cases and controls, while alcohol consumption was more prevalent among controls. The proportion of individuals with a GC family history or *H. pylori *infection, however, was more common among cases [3].

Genotype distributions for controls were consistent with HWE, and the average call rate for the analysed SNPs was 97.5% (range: 95.6% to 98.7%).

We observed no evidence for significant associations of the eleven SNPs and CAG risk (Additional file 1). For each SNP, similar ORs were obtained with modified serological definitions in sensitivity analyses (data not shown). Neither did the analyses stratified by the age groups < and ≥ 65 years show statistically significant association between any SNP and CAG (Additional file 2).

## Discussion

To our knowledge, the present investigation, nested within the population-based German ESTHER cohort, is the largest study addressing genetic susceptibility to CAG [3,21].

Despite both sample size and rationales, we did not find consistent associations between DNA repair SNPs and risk of CAG, the well-established precursor of intestinal GC (Additional files 1 and 2). In contrast, Capellá et al. found associations of ERCC2/XPD D312N and K751Q with an increased risk of severe CAG [20]. The discrepant findings may be due to different serological definitions of CAG in the studies [3,20]. Another possible reason to be considered is random variation, having in mind the much smaller number of cases (n = 246) in the study by Capellá et al. [20].

Remarkably, *XRCC1 *-77T > C was identified as functional polymorphism, diminishing promoter activity and thus increasing the risk of non-small cell lung cancer (NSCLC), while the three non-synonymous XRCC1 SNPs R194W, R280H and R399Q, whose functional characteristics are not determined yet, showed no association with NSCLC risk [15]. As demonstrated by Capellá et al., R399Q in XRCC1 showed an association with an increased risk of severe chronic atrophic gastritis [20]. Contrariwise, a recent meta-analysis indicated R194W to be XRCC1 susceptibility variant for GC [23]. After extensive studies of DNA repair polymorphisms in various cancer sites and ethnic populations, the results still remain inconsistent [17], which may be attributed to both different aetiologies of cancers, and ethnic or geographical disparities [11].

The present study has both strengths and limitations. Strengths include the well-defined and homogeneous study population. In addition, we analysed SNPs for which associations with CAG (and/or GC and other cancers) are biologically plausible and for which associations have been previously reported [9-20]. We had a power of 80% at a significance level of 0.05 to detect ORs ≥ 1.44/≤ 0.69 (ranges 1.37-1.44/0.73-0.69) for all SNPs [22]. Admittedly, the presented data need to be interpreted within caution as the best serological definition of CAG is difficult to accomplish. Thus, Miki et al. suggested to use the PG I/PG II ratio for the definition of CAG and reported PG I alone to be specific, yet insufficiently sensitive [24], and a series of studies agreed to the necessity to include the PG I/PG II ratio in the definition [25,26].

Although the eleven investigated SNPs are strong candidates for susceptibility to cancers and their precursor lesions [5-16], this study did not indicate any major association with CAG risk. A possible explanation for the lack of significance may be that a real risk altering SNP (within one of the selected or another DNA repair pathway gene) was not analysed and missed.

## Conclusions

Our findings, based on a large number of CAG cases, do not support associations of DNA repair pathway gene SNPs with the risk of CAG. Much larger studies are needed to reveal potential weak associations. Moreover, full coverage of candidate DNA repair genes, i.e. tagging SNP approaches should be aimed for in future studies.

## Competing interests

The authors declare that they have no competing interests.

## Authors' contributions

BF, HB, HM, MNW, ER were responsible for the study design. BF, NK, TI were involved in data acquisition and analysis. Statistical analyses, data interpretation and manuscript drafting were done by BF. All authors critically reviewed and approved the final manuscript.

## Pre-publication history

The pre-publication history for this paper can be accessed here:

http://www.biomedcentral.com/1471-2407/11/440/prepub

## Supplementary Material

Additional file 1 **Table S1**. DNA repair pathway single nucleotide polymorphisms (SNPs) and risk of chronic atrophic gastritis. Click here for file

Additional file 2 **Table S2**. Associations of DNA repair pathway single nucleotide polymorphisms (SNPs) with the risk of chronic atrophic gastritis among individuals < and ≥ 65 years of age. Click here for file
