# Supplementary material for: DNA repair gene polymorphisms and risk of chronic atrophic gastritis: a case-control study
Source: BMC Cancer. 2011 Oct 11;11:440. doi: 10.1186/1471-2407-11-440 (PMC3209461; doi:10.1186/1471-2407-11-440)
Supplement: Additional file 1 — Table S1. DNA repair pathway single nucleotide polymorphisms (SNPs) and risk of chronic atrophic gastritis. [file 1471-2407-11-440-S1.DOC]

**Table 1.** DNA repair pathway single nucleotide polymorphisms (SNPs) and risk of chronic atrophic gastritis

CI, confidence interval; ID, identification; OR, odds ratio. ^a^ORs were adjusted for age and sex.
