# Supplementary material for: DNA repair gene polymorphisms and risk of chronic atrophic gastritis: a case-control study
Source: BMC Cancer. 2011 Oct 11;11:440. doi: 10.1186/1471-2407-11-440 (PMC3209461; doi:10.1186/1471-2407-11-440)
Supplement: Additional file 2 — Table S2. Associations of DNA repair pathway single nucleotide polymorphisms (SNPs) with the risk of chronic atrophic gastritis among individuals < and ≥ 65 years of age. [file 1471-2407-11-440-S2.DOC]

**Table 2.** Associations of DNA repair pathway single nucleotide polymorphisms (SNPs) with the risk of chronic atrophic gastritis among individuals < and ≥ 65 years of age

CI, confidence interval; ID, identification; OR, odds ratio.

^a^ORs were adjusted for sex.
